# Allelic Diversity of Major Histocompatibility Complex Class II DRB Gene in Indian Cattle and Buffalo

**DOI:** 10.4061/2011/120176

**Published:** 2011-01-27

**Authors:** Sachinandan De, Raj Kumar Singh, Biswajit Brahma

**Affiliations:** ^1^Animal Biotechnology Centre, National Dairy Research Institute, Karnal 1, Haryana 23001, India; ^2^National Research Centre on Equines, Sirsa Road, Hisar 1, Haryana 25001, India; ^3^KVK, SKUAST-Jammu, Bhaderwah, Jammu 182221, India

## Abstract

The present study was conducted to study the diversity of *MHC-DRB3* alleles in Indian cattle and buffalo breeds. Previously reported *BoLA-DRB* exon 2 alleles of Indian Zebu cattle, *Bos taurus* cattle, buffalo, sheep, and goats were analyzed for the identities and divergence among various allele sequences. Comparison of predicted amino acid residues of *DRB3* exon 2 alleles with similar alleles from other ruminants revealed considerable congruence in amino acid substitution pattern. These alleles showed a high degree of nucleotide and amino acid polymorphism at positions forming peptide-binding regions. A higher rate of nonsynonymous substitution was detected at the peptide-binding regions, indicating that *BoLA-DRB3* allelic sequence evolution was driven by positive selection.

## 1. Introduction

Major histocompatibility complex (MHC) class I and class II are cell surface molecules that play an important role in intercellular recognition and self/nonself discrimination and trigger humoral and cell-mediated immune responses [1]. MHC class II molecules are heterodimeric glycoproteins and are composed of two noncovalently associated *α* and *β* chains expressed on macrophage, B-cell, and other antigen processing cells. MHC class I molecules present endogenous peptide antigen to cytotoxic (CD8+) T-cell, whereas class II molecules present exogenous antigen to helper (CD4+) T-cell to generate immune response [2]. 

Genes encoding MHC molecules are the most polymorphic genes described in vertebrates with polymorphism occurring predominantly at peptide-binding sites [3]. There is growing evidence for an association between MHC types and susceptibility to pathogens [4, 5]. *MHC* genes in bovines (bovine lymphocyte antigen; *BoLA*) have been mapped on chromosome 23 (BTA23) and *DR* and *DQ *have been identified as the two-principal class II molecule in ruminants including cattle. They are located in class IIa cluster and are tightly linked with class III and class I genes [6]. In *BoLA-DR* subregion of cattle, at least three different *DRB* loci have been described along with pseudogene and gene fragments [7]. However, *DRA* and *DRB3* have been found as major expressed gene pair [8]. *DRB3* (*BoLA-DRB3*) has been found to be highly polymorphic and is responsible for the differences in the susceptibility to infectious disease. *DRB1* is a pseudogene and *DRB2* gene is transcribed at very low levels in lymphocyte tissue [9, 10]. Polymorphism of *BoLA-DRB3* is confined mainly to second exon that encodes for *β*1 domain, responsible for peptide-binding sites. Recent *BoLA *databases (http://www.projects.roslin.ac.uk/bola/bolahome.html, http://www.ebi.ac.uk/cgi-bin/ipd/mhc/view_nomenclature.cgi?bola.drb3) suggest more than 100 alleles of *DRB3* gene in *Bos taurus* and *Bos indicus* cattle. Various alleles of this locus are found to be associated with the progression of infectious diseases [11, 12]. Compared to other ruminant species, buffalo MHC locus has been less extensively studied. MHC gene complex of buffalo has been mapped on chromosome 2 [13] but very few reports are available on their nature of diversity [14, 15]. Indian Zebu cattle and buffaloes are well adapted to tropical climate and are resistant to many common infectious diseases. Therefore, the present study was aimed to analyze the variations of allelic forms of MHC-DRB exon of cattle and buffalo and compare the variation with other ruminant species. 

## 2. Methods

### 2.1. Source of Sequence

For comparative sequence analysis, we included our previously reported MHC-DRB Exon II sequences of zebu cattle (AF261953-AF261954, AF272862–AF272876, AF272878–AF272882) and buffalo (AF3854473–AF385480, AF261955-AF261956, AF270653–AF270659, AF270661–AF270673) [14, 16]. Besides, other reported *BoLA-DRB3* sequences from official *BoLA* web page (http://www.projects.roslin.ac.uk/bola/bolahome.html, http://www.ebi.ac.uk/cgi-bin/ipd/mhc/view_nomenclature.cgi?bola.drb3) and *DRB* sequences from other ruminant species like sheep (*Ovis aries*, AF126432–AF126441, Y10245–Y10249, U00204–U00219, U00221–U00225, U00227–U00237), goat (*Capra hircus*; AB008347–AB008362), big horn sheep (*Ovis canadensis*; AF324–AF324861), white-tailed deer (*Odocoileus virginianus*; AF082161–AF082175), and red deer (*Cervus elaphus*; U11101–U11108, U11110–U11116, U11118–U11119, U11121, U11212, U11213, and U11215–U11218) were included in the analysis. The BLAST algorithm was used to search the GenBank databases (http://www.ncbi.nlm.nih.gov/) for homologous sequences. Sequence editing and translations were carried out using *Sequence Manipulation Suite* version 2.0 [17]. Multiple alignments of the nucleotide and amino acid sequences were carried out by the CLUSTAL-W multiple sequence alignment programme [18]. Identical sequences were removed to get a total of 90 sequences for cattle (*BoLA-DRB3*), 55 for sheep, 20 for goat, 24 for red deer, 21 for big horn sheep, and 15 for white-tailed deer. All sequences were edited to get a uniform length of 234 bp nucleotides per sequence before analysis. 

### 2.2. Sequence Analysis

To determine the identities and divergence among various alleles, sequences were aligned by the Genedoc [19]. The phylogenetic analysis was performed using the Molecular Evolutionary Genetics Analysis (MEGA 3.0) software [20]. Amino acid sequences responsible for the peptide-binding sites were identified by comparison with the peptide-binding structure of human DR molecule [3]. Relative frequencies of non-synonymous (*dN*) and synonymous (*dS*) substitutions with standard errors for the peptide-binding sites (PBS) and non-PBS were calculated by the Nei and Gojobori (1986) [21] method and using the Jukes and Cantor (1969) [22] correction incorporated in MEGA 3.0 [20] Their standard errors were obtained through 1000 bootstrap replicates. The significance of the difference between these synonymous and non-synonymous substitution rates wase tested statistically with a Z-test of selection at the 5 percent level, whereby the *P*-values were the probability of rejecting the null hypothesis of positive selection (*dN/dS*) [20]. 

The phylogenetic tree was constructed using the Neighbor-Joining method [23]. The evolutionary distances were computed using the Poisson correction method [24]. All positions containing gaps and missing data were eliminated from the dataset (complete deletion option). The resulting trees were evaluated by bootstrap analysis [25] based on 1000 resamplings. 

## 3. Results

The synonymous and nonsynonymous substitution of nucleotides and amino acids of DRB locus in bovine and related ruminant species is presented in Table 1. The pairwise comparisons between all the DNA sequences showed an identity ranging from 86 to 97 percent within total 22 alleles of *BoLA-DRB3 *gene of Indian Zsebu cattle. A total of 60 out of 234 (25.64%) nucleotides (alignments not shown here) and 27 of 78 (34.61%) amino acids were variable. Substitutions of amino acids tended to be clustered around sites, postulated to be responsible for selective peptide recognition regions (PBR) [3]. Twenty two of 78 (28.20%) amino acid sites belonged to the putative PBR. Of these, 16 (72.72%) were polymorphic. In contrast, 11 out of 56 (19.64%) non-PBR sites were variable. Within the PBR, the rate of nonsynonymous substitutions (dN = 0.332 ± 0.064) was higher than that of synonymous substitutions (dS = 0.139 ± 0.050). However, for non-PBR codons, dN  (0.074 ± 0.018) value was little higher than dS  (0.055 ± 0.018). In almost all ruminant species analyzed here, the frequency of non-synonymous substitutions (*dN*) was comparatively higher than that of synonymous substitution (*dS*) in the putative PBR (Table 1). In the non-PBR region, the non-synonymous substitution was comparatively higher than the synonymous substitution. The high ratio (2.38) of non-synonymous to synonymous substitutions (*dN/dS*) indicates strong positive selection for diversity at the PBR. A still higher value for *dN/dS* ratio was also estimated in case of goat (4.13), sheep (5.43), white-tailed deer (5.36) and big horn sheep (8.9). The value was found lower in case of river buffalo (2.3).

The sharing of DRB polymorphism at the amino acid level found in other ruminants is presented in Table 2. Most variability was found in amino acid residues 11, 13, 28, 32, 37, 56, 57, 59, 60, 61, 67, 70, 71, 74, and 86. In bovine, amino acid residues at positions 11 and 37 were highly polymorphic with seven amino acids per site. However, residues at 12, 30, 45, and 48 were selectively polymorphic than other ruminants like sheep, goat, buffalo, red deer, white-tailed deer, and big horn sheep (Table 2). The amino acids for other polymorphic sites were common in most of the species. The level of polymorphism was the highest in cattle and followed by sheep and goat.

**Figure 1:**
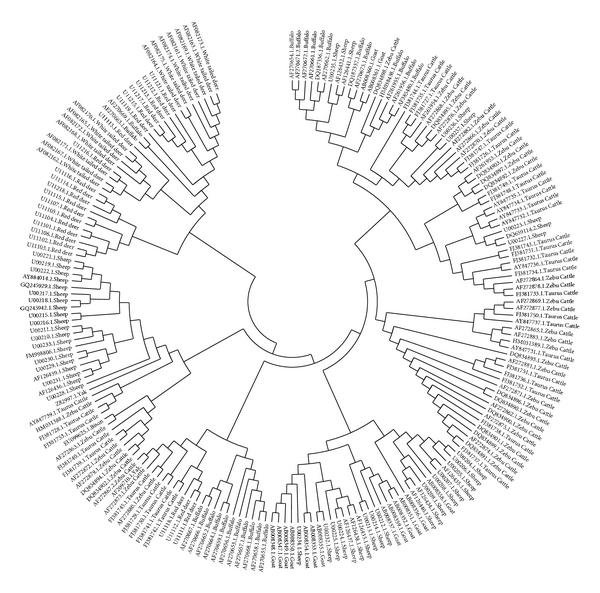
The phylogenetic tree showing evolutionary relationship of MHC class II DRB gene of different taxa. The optimal tree with the sum of branch length = 8.88963793 is shown.

The phylogenetic relationship tree involving sequences of MHC class II DRB gene of different species has been shown in the dendrogram (Figure 1). The tree depicted several clades based on the similarity in the amino acid residues present in the selected region. Along with the species-specific clade few mixed branches were also visible in the tree. Red deer, cattle and buffalo alleles were found to be clustered in respective separate places showing their uniqueness in the DRB alleles. Sheep and goat alleles were represented together in a single clade. In one end of the tree, there was one distinct mixed clade representing cattle, buffalo sheep and goat DRB alleles. In Zebu cattle (Bos indicus) and Taurine cattle (Bos taurus) alleles were represented together. Two yak alleles and one bison allele were located in the cattle clade representing their closeness to cattle alleles. Still a more number of alleles from each species might still give more comparative picture of this DRB allele diversity pattern. 

## 4. Discussion

Comparison of predicted amino acid residues of *DRB3* exon 2 alleles with similar alleles from other ruminants revealed considerable congruence in amino acid substitution pattern (Table 2). Extensive polymorphism was revealed in the peptide-binding amino acid region. Out of all peptide-binding sites, in position 37 seven different amino acids were encountered followed by six amino acids in the position 11. In the non peptide-binding region position 57 and 67 were found to be highly variable containing four amino acid substitutions. The allelic nucleotide sequence divergence (*d*) was found up to 12.3 percent (K2P distance; see Table 1). The rate of amino acid substitution was compared for peptide-binding and non-binding region. The high ratio of non-synonymous substitution to synonymous substitution was found in the PBR (Table 1). This high ratio of dN/dS indicates that non-synonymous sites evolved faster than synonymous sites and implies balancing selection (or positive Darwinian selection) favored new variants and increased allelic polymorphism [26, 27]. The ratio was even higher when only putative peptide-binding sites were considered. The pattern and level of *DRB3 *gene polymorphism revealed in the present study could be a consequence of adaptation to Indian hot and humid climate with a relatively high level of exposure to pathogens. However, due to small sample size in the present analysis, it is difficult to recommend any conclusion on *DRB3* variability in Indian population. Moreover, the five populations sampled were not complete representative of the species distributed across India. There could be much higher variability that exists in many other breeds and locations. 

The polymorphism at *DRB* loci of many artiodactyla species has been reported by many workers. Among these, high polymorphism has been found in Alpine chamois (*Rupicapra rupicapra*), goat (*Capra hircus*), big horn sheep (*Ovis canadensis*), white-tailed deer (*Odocoileus virginianus*) and red deer (*Cervus elaphs*). In our study, dN/dS ratios were on higher side for big horn sheep, sheep, white-tailed deer, and goat. Comparatively lower values were observed for red deer and cattle. Limited polymorphism has been reported in other ruminants like roe deer (*Capreolus capreolus*) and reindeer (*Rangifer tarandus*). Some species like musk ox (*Ovibos moschatus*) and fallow deer (*Cervus dama*) have been found monomorphism for the DRB locus [28]. Sharing of allele could not be found among all these ruminants. However, certain alleles were found to be closely related between species. This indicates that these alleles had separated from their common ancestor more than 1.5 million years ago [29]. 

Cattle, sheep and goat, in spite of their early domestication process, represented many *DRB* alleles with high heterozygosities and intermediate to large genetic distances between them [28]. This indicated their higher adaptability supported by very large effective population size spread over different geographical regions. It was reported that polymorphism of MHC genes was driven by a strong balancing selection mechanism [27]. This is represented by their higher values of dN : dS ratio. More populations have to be extensively surveyed and exact MHC-peptide interaction has to be evaluated for each allele to explore exact significance of this higher polymorphism in these Indian breeds. 

## Figures and Tables

**Table 1:** Comparison of average rate of nonsynonymous (dN) and synonymous (dS) substitutions with standard errors obtained through 1000 bootstrap replicates in parentheses for the peptide-binding region (PBR) and non-peptide binding region (non-PBR) and their ratio among different *BoLA-DRB3* alleles. *N*: number of codons.

Alleles	No alleles	Positions	*N*	*d* (K2p)	*dN* (SE)	*dS* (SE)	*dN/dS*	*P*
Indian cattle	22	PBR	22	0.283(0.042)	0.332(0.064)	0.139(0.050)	2.38	.008
		Non-PBR	56	0.069(0.012)	0.074(0.018)	0.055(0.018)	1.34	.211
		All	78	0.123(0.014)	0.137(0.022)	0.076(0.019)	1.80	.009
*BoLA *alleles	89	PBR	22	0.256(0.041)	0.311(0.072)	0.102(0.039)	3.05	.003
		Non-PBR	56	0.035(0.008)	0.039(0.013)	0.019(0.013)	2.05	.140
		All	78	0.090(0.012)	0.105(0.021)	0.041(0.014)	2.56	.002
Sheep	55	PBR	22	0.176(0.033)	0.223(0.062)	0.041(0.025)	5.43	.001
		Non-PBR	56	0.042(0.009)	0.045(0.013)	0.037(0.014)	1.21	1.000
		All	78	0.077(0.011)	0.087(0.018)	0.045(0.014)	1.93	.019
Goat	20	PBR	22	0.218(0.038)	0.269(0.067)	0.065(0.031)	4.13	.001
		Non-PBR	56	0.058(0.011)	0.062(0.015)	0.046(0.017)	1.34	.200
		All	78	0.099(0.013)	0.114(0.019)	0.050(0.014)	2.28	.002
Buffalo	28	PBR	22	0.238(0.039)	0.277(0.067)	0.120(0.064)	2.30	.025
		Non-PBR	56	0.079(0.013)	0.065(0.014)	0.126(0.031)	0.51	1.000
		All	78	0.118(0.014)	0.127(0.021)	0.097(0.020)	1.30	1.000
Big horn sheep	21	PBR	22	0.224(0.042)	0.294(0.073)	0.033(0.018)	8.90	.000
		Non-PBR	56	0.045(0.010)	0.046(0.012)	0.044(0.020)	1.04	.470
		All	78	0.090(0.013)	0.106(0.019)	0.041(0.015)	2.58	.005
White tailed deer	15	PBR	22	0.254(0.046)	0.327(0.079)	0.061(0.028)	5.36	.000
		Non-PBR	56	0.062(0.011)	0.065(0.015)	0.054(0.021)	1.20	.286
		All	78	0.110(0.014)	0.128(0.023)	0.055(0.016)	2.32	.002
Red deer	24	PBR	20	0.235(0.038)	0.282(0.068)	0.108(0.045)	2.61	.007
		Non-PBR	55	0.064(0.011)	0.067(0.016)	0.055(0.022)	1.21	.126
		All	75	0.105(0.013)	0.117(0.022)	0.068(0.019)	1.72	.003

**Table 2:** Interspecies comparison of polymorphic amino acid substitutions for DRB exon 2 molecules in cattle, sheep, goat, buffalo, red deer, white-tailed deer and big horn sheep. The bottom row indicates the number of sequences examined in each species (*in the amino acid position column indicates that this position forms part of the peptide binding groove according to the model of Brown et al. 1993).

Codon position	Alleles of Indian cattle	BoLA alleles	Sheep	Goat	Buffalo	Red deer	White-tailed deer	Big horn sheep
*09	EQ	EQ	E	ER	EQ	EL	ESV	E
10	Y	Y	Y	Y	Y	HQY	HY	Y
*11	ACHLST	ACHLRSYT	AHRSYT	CHSTY	ARSVY	AFHLPST	AFGHP	AHRTY
12	KT	KQT	KRT	KT	KT	KT	K	K
*13	GKRS	GKRS	KRS	AGKRS	GKRS	AGRS	AGKS	GKS
14	E	E	E	E	EG	E	E	E
15	C	C	C	C	C	C	C	C
16	H	H	HR	H	H	HPY	H	HR
17	F	F	F	F	F	F	F	F
18	F	F	FS	FS	FS	FPS	S	FS
19	N	DN	N	N	N	N	N	N
20	G	G	G	G	G	G	G	G
21	T	T	T	T	T	T	T	T
22	E	E	E	EGQ	E	EQ	EQ	E
23	R	R	R	R	R	R	R	R
24	V	LV	V	V	V	MV	V	V
25	R	QR	RW	GRW	QRW	GQR	QR	R
26	FLY	FLY	FLY	FLY	FLY	FLSY	FLY	FLY
27	L	L	L	L	LR	L	L	L
*28	DEH	DEHN	DE	DH	DEQ	ADEGQ	DEFQV	DEH
29	R	R	R	R	GR	R	R	R
*30	CHSY	CHSY	Y	Y	HY	Y	DY	FY
31	FY	FY	FY	FY	FIY	FIV	FIV	FY
*32	HTY	HTY	HTY	HTY	STY	CHY	Y	HTY
33	N	N	N	N	N	KNS	N	N
34	G	G	G	G	G	EGRW	GKQR	G
35	E	E	E	E	EK	E	E	E
36	E	E	E	E	E	EG	E	E
*37	FHLNRTY	FHLNRTY	FNTY	FINTY	LNTY	FY	FIY	FNTY
*38	AV	AV	ALV	LV	LV	AV	LV	AV
39	R	R	R	R	TR	R	R	R
40	F	F	F	FY	F	FY	FY	F
41	D	D	D	D	D	D	D	D
42	S	S	NS	NS	GS	CS	S	NS
43	D	D	D	D	D	D	DN	D
44	W	W	W	RW	QRW	VW	V	W
45	DG	DGS	GS	G	G	G	G	G
46	E	E	E	E	E	EQ	E	E
*47	FY	FY	FY	FY	FY	FY	FY	FY
48	QR	QRW	R	R	R	QR	R	R
49	A	AE	AP	A	A	A	A	A
50	V	LV	V	V	LV	V	V	AV
51	T	T	AT	AT	T	T	T	AT
52	E	E	E	E	E	EK	E	EQ
53	L	L	L	LQ	L	L	L	LQ
54	G	G	G	G	G	G	G	G
55	RW	PQR	R	RQ	PQR	R	QR	QR
*56	PR	PQR	PQR	EPQ	PQ	P	PT	EPR
57	ADSV	ADSV	AEDS	DENST	DIS	DSV	DES	ADS
58	A	AR	AV	A	A	A	A	A
59	EKV	EKV	EK	EK	EK	EK	DEK	EK
*60	HQY	HLQY	HQY	Y	DLQY	HY	DGNY	HY
*61	LW	CLW	W	W	W	LWY	FWY	W
62	N	N	N	N	N	N	N	N
63	GS	GS	S	S	GS	S	RS	S
64	Q	Q	Q	Q	Q	LQR	QR	Q
65	K	K	K	K	EK	K	K	K
66	DE	DE	DEN	DE	DG	E	DE	DE
67	FILT	FILT	FIL	FIL	FI	ILY	FIL	FIL
68	L	L	L	L	L	LM	L	L
69	E	E	E	E	E	E	E	E
*70	DEQR	DEQR	QRS	DQNRS	DQR	DEQR	DEQRY	QRS
*71	AEKR	AEK	AKRT	KRS	KQRS	AEHKLNRT	KLNRSW	AKRT
72	R	R	R	R	R	GR	R	R
73	A	A	APT	AT	AW	A	AT	A
*74	AENSY	AENSY	AENT	AENS	AENST	AEN	AELS	AEN
75	V	V	V	AV	V	V	AV	V
76	D	D	DN	D	D	D	D	D
77	RT	RT	T	KT	**AGKRST**	RT	T	RT
*78	VY	VY	VY	FVY	FVY	FVY	VY	VY
79	C	C	C	C	C	C	C	C
80	R	R	R	R	R	R	IRS	R
*81	H	HY	H	HY	HY	HY	H	HR
*82	N	N	N	DNY	N	DN	N	N
83	Y	Y	Y	Y	Y	Y	SY	Y
84	G	G	G	G	GR		G	G
*85	GV	GV	V	V	V		IV	V
*86	GV	FGMV	FGID	FGILV	DFGV		FGI	FGIV
	*n* = 22	*n* = 89	*n* = 55	*n* = 16	*n* = 28	*n* = 24	*n* = 15	*n* = 21
